# The Efficiency of Probiotics Administrated via Different Routes and Doses in Enhancing Production Performance, Meat Quality, Gut Morphology, and Microbial Profile of Broiler Chickens

**DOI:** 10.3390/ani11123607

**Published:** 2021-12-20

**Authors:** Elham A. Soumeh, Astrid Del Rocio Coba Cedeno, Shahram Niknafs, Jacoba Bromfield, Louwrens C. Hoffman

**Affiliations:** 1 School of Agriculture and Food Sciences, Gatton Campus, The University of Queensland, Gatton, QLD 4343, Australia; a.cobacedeno@uq.net.au (A.D.R.C.C.); Jacoba.Bromfield@bioproton.com (J.B.); 2 Queensland Alliance for Agriculture and Food Innovation, The University of Queensland, St Lucia, QLD 4072, Australia; s.niknafs@uq.edu.au (S.N.); louwrens.hoffman@uq.edu.au (L.C.H.); 3 Bioproton Pty Ltd., Acacia Ridge, Brisbane, QLD 4110, Australia; 4 Department of Animal Sciences, Stellenbosch University, Stellenbosch 7906, South Africa

**Keywords:** antibiotics, broiler chicken, growth performance, gut morphology, nutrient digestibility, probiotics in feed or water

## Abstract

**Simple Summary:**

Antimicrobial growth promoters (AGPs) have been used in the animal production industry around the world for decades, with the consequence of a high potential of antibiotic-resistant bacteria transfer to humans. Efficiently raising broiler chickens in an antibiotic-free production system is a challenge, and identifying an effective nutritional alternative to support growth performance, gut health, and functionality without administrating AGPs is of essence. Several antimicrobial alternative options that are commercially available include herbal essential oils, exogenous enzymes, organic acids, plant secondary metabolites, probiotics, and prebiotics. Probiotics in animal feed is projected to attain a massive global growth, reaching USD 6.24 billion by 2026. This study tested the efficiency of probiotics when supplemented via different administration routes (feed or water) and doses, or in combination with prebiotics, on growth performance, meat quality, gut morphology, and microbial profile of broiler chickens. The outcomes revealed that probiotics enhance production performance, and compared to AGPs, do not reduce the beta-diversity of the gut microbial community. Water-soluble probiotics seemed to be more effective in improving growth performance.

**Abstract:**

To study the efficiency of *Bacillus* spp. probiotics administered via different routes and doses, a 6-week grow-out trial was conducted using a total of 378 day-old mixed-sex ROSS308 broiler chickens in a completely randomized block design. Six experimental diets included probiotics added at two different inclusion rates into the feed (250 g/ton; PRO250, or 500 g/ton; PRO500), or in the drinking water (25 g/L; PRO-WS), or as a feed synbiotic (250 g probiotic + 250 g/ton prebiotic; SYN), compared to a negative (NC; without additives) and positive control (PC; with antibiotics) diets. The PRO-WS enhanced feed intake (*p* < 0.05) and tended to improve average daily gain and final body weight (*p* = 0.14). Broiler gut morphology in the duodenum including the villus height (*p* = 0.04), villus width (*p* = 0.05) and crypt depth (*p* = 0.02) were improved by PRO500. Firmicutes was the most abundant phylum, followed by Bacteroidetes. *Streptococcaceae, Lachnoospiraceae, Peptostreptococcaceae, Ruminococcaceae*, and *Erysipe-lotrichaceae* were the top five most abundant families. Antibiotic inclusion in PC reduced microbial beta-diversity and increased similarity compared to probiotic inclusion (*p* = 0.05). Probiotic inclusion reduced the relative abundance of *Bacteroides fragilis*, which is a commonly isolated pathogen and is considered as a marker for antimicrobial resistance. Overall, probiotic supplementation via feed or water may potentially improve the production performance of the broiler chickens, and water-soluble probiotics are potentially more effective. Probiotics, especially when added to water, suggest a promising feed additive to support gut microbial maturation and diversity, and may reduce resistant bacteria in broiler chickens. However, it is suggested that the best route for the administration of probiotics be further examined under commercial conditions to find the most effective and practical application method that yields the most consistent results.

## 1. Introduction

According to OECD-FAO Agricultural Outlook 2020–2029, global chicken meat consumption is projected to increase to 145 million tons by 2029, thereby accounting for 50% of the increased global meat consumption [1]. The intensification of meat chicken farming has led to an increase in the use of antibiotics to improve growth performance and control the spread of diseases. The prolonged use of antibiotics at subtherapeutic levels has resulted in bacterial resistance and accumulation of residues in meat products having detrimental effects on animal and human health [2]. Therefore, multiple countries have banned subtherapeutic use of antibiotics as a growth promoter in animal production resulting in the search for alternative practices to support healthy and efficient chicken production in an antibiotic-free system. Probiotics are mono- or mixed-cultures of live beneficial bacteria which have been used in poultry production to establish a healthy and diverse gut microbial ecosystem which competitively excludes pathogens [3]. The use of commensal bacteria induces an immune response in poultry through fortification of the mucosal barrier and stimulating innate immune responses, thereby enhancing growth performance and nutrient digestibility [4,5,6]. Prebiotics are the non-digestible fermentable fraction of feed ingredients, mostly natural oligosaccharides, and/or small sugar molecules, and/or soluble fibre fractions which selectively stimulate the growth and activity of the beneficial gut bacteria [7,8]. Synbiotics, a combination of probiotics and prebiotics, provide the beneficial bacteria together with nourishing material thereby improving the survival and establishment of the directly fed microbes in the gastrointestinal tract [9,10]. Different routes of administrating probiotic preparations to the broiler chickens are used, including via feed, water, gavage including droplet or inoculation, spray, or litter [11]. Although feed supplementation is the most used route of probiotic administration in poultry production, the survival rates of the bacteria in pelleted diets are low, although spore-forming bacteria strains have higher stability to heat, acidic pH, and the harsh environments typically encountered during the pelleting procedures [12,13]. The water-soluble probiotics are the next generation of probiotics with a new formula, ensuring a quick and homogeneous distribution and release of the probiotic spores in water and have been reported to be more efficient in improving broiler chickens’ growth performance [4,14]. The overall number of bacteria in the gastrointestinal tract (GIT) exceeds that of the host body’s eukaryotic cells. There are three types of bacteria in the host: dominant bacteria (more than 10^6^ CFU/g sample), subdominant bacteria (10^3^ to 10^6^ CFU/g sample), and residual bacteria (less than 10^3^ CFU/g sample) [15]. From the crop to the lower ileum, the poultry GIT is dominated by Gram-positive, mostly facultative anaerobes, whilst the ceca are dominated by Lactobacillus, Enterococcus, coliforms, and yeasts. Low pH induces bacterial population reduction in the proventriculus and gizzard, where in the duodenum, enzymes, high oxygen pressure, and bile salts reduce the microbial concentration. The conditions of the lower small intestine and large intestine are suitable for the formation of diverse microbiota. Establishing a healthy diverse gut microbiota plays a protective role as the first line of defence against pathogenic bacteria, enhances the gut structure integrity, and assists in specific metabolic activities [16]. It is reported that the bacterial communities in the hindgut are dynamic and change or diversify with diet and age [17]. The current study aimed to compare the efficiency of probiotics administration added at two different inclusion rates into the feed, or as a water-soluble probiotic in the drinking water, or as a feed synbiotic in combination with prebiotics, compared to negative (without additives) and positive control (with antibiotics) diets.

## 2. Materials and Methods

All experimental procedures, including animal handing and husbandries, were approved by the Animal Ethics Committee of Research Ethics Unit of The University of Queensland, AEC Approval Number SAFS/579/18.

### 2.1. Birds, Diets and Experimental Design

A total of 378 1-day old mixed gender broiler chickens (ROSS 308) were purchased from a commercial hatchery (Woodlands Hatchery, QLD) and transferred to the Queensland Animal Science Precinct (QASP) facility at the University of Queensland (Gatton Campus, Qld 4343, Australia). Birds were vaccinated for Marek’s disease and infectious bronchitis before transportation. On arrival at the facilities, all birds were weighed individually and distributed randomly into 54 floor pens. Pens were randomly assigned to one of six experimental groups in a completely randomized block design (CRBD), with nine replicate pens per experimental group and seven birds in each (*n* = 63 chickens per experimental group).

A basal wheat–corn–soybean meal diet was purchased from a commercial feed miller, Allora Grains & Milling (Down Holdings Pty Ltd, Ellinthrope QLD 4362, Australia) and transported to the facility. The feed-supplemented probiotic and synbiotic products were added to the basal diet in the facility prior to the start of the experiment. The experimental treatments (Table 1) included a standard mash wheat–corn–soybean diet (Negative Control; NC); NC + 200 g/t Virginiamycin (Positive Control; PC); NC + 250 g/t Probiotic (PRO250); NC + 500 g/t Probiotic (PRO500); NC + 0.25 g/L Probiotic in Water (PRO-WS); and NC + 250 g/t probiotic + 250 g/t prebiotic (SYN). The probiotic added to all experimental diets was Natupro® and the water-soluble probiotic was Natupro W® (supplied by Bioproton Pty Ltd (Acacia Ridge, Queensland 4110, Australia). Natupro® contains a collection of multiple Bacillus strains (Table 2) and the prebiotic, a commercial yeast cell wall (YCW) product containing 20% β-glucan and 20% mannan-oligosaccharides (MOS), the latter with the commercial name of X-MOS supplied by Algebra Bio Pty Ltd (Balmain, NSW 2041, Australia).

The PRO-WS was made twice per week to be served to the birds as fresh as possible. Two tanks of 40 L were used to make the solution and the pens on the PRO-WS treatment, had bell drinkers (5 L) filled from the tank containing the PRO-WS. Each replicate group of broiler chickens was reared in floor pens (120 × 120 × 80 cm) with carboard beddings for a total of 42 days and had ad libitum access to feed and water for the entire trial period. The grow-out period was divided into three phases (starter: day 1–14; grower: day 14–28; finisher: day 28–42), and nutrient levels were adjusted accordingly as per ROSS 308 guidelines (Table 3). Calculated and analysed nutrient composition of experimental diets (as-is) is presented in Table 3. Throughout the trial, the ROSS 308 management guidelines were followed to meet the broiler nutrient recommendations and the appropriate environmental conditions including temperature, relative humidity, and lighting [18]. The lighting program provided 23 h of light at a 30–40 lux intensity and 1 h of dark (less than 0.4 lux) for the first 7 days and a minimum of 4 h darkness and a light period of 10 lux intensity after 7 days. Temperature was set at 32 °C and 40% relative humidity for the first 7 days and a 2 °C reduction per week after 7 days until the temperature reached 24 °C at 27 days and 40% relative humidity. This temperature and relative humidity were maintained until the end of the trial. Each pen (except for the PRO-WS replicates) had two nipple drinkers adjusted weekly to the birds’ height and one cone feeder.

### 2.2. Chemical Analyses

The experimental diets were analysed for dry matter (DM), total nitrogen (N), and ash contents following the Association of Official Agricultural Chemists (AOAC) (2005). Dry matter content was determined by drying the sub-samples in an oven at 105 °C for 24 h. Total N content of feed samples was analysed using a LECO CNS928 carbon/nitrogen combustion analyser 1.0 (Leco, St. Joseph, MI, USA) following the instructions of the manufacturer, and the crude protein (CP) was calculated (6.25 × N). 

### 2.3. Growth Performance

Body weight (BW) of individual birds and feed intake (FI) of each replicate pen were recorded weekly and average daily gain (ADG), average daily feed intake (ADFI) and feed conversion ratio (FCR: feed intake/live weights of birds per replicate) were calculated.

Chicken mortality was recorded daily during morning and afternoon inspection and used for chicken-day calculations. The feed intake and FCR were corrected for mortality accordingly. 

### 2.4. Slaughtering, Carcass Composition, and Sample Collection

On the last day of the trial (42 days), 1 bird per replicate was arbitrarily chosen, weighed and euthanised by electrical stunning (240 V for 10 s) and exsanguination to collect blood samples. The carcass was then soaked in a scalding tank for 2 min (water temperature 60 °C) followed by defeathering and evisceration. Dressed carcass weights were recorded before dissection followed by recording the removed internal organs’ weights (heart, liver, proventriculus, gizzard, spleen, bursa of Fabricius, pancreas, and abdominal fat pad). The relative weights of individual organs were calculated in ratio to final body weight. Additionally, the small intestine of each bird was sampled to study gut pH and histomorphology. After the evisceration process, the birds’ carcasses were individually weighed before and after chilling at 4 °C for 24 h before commencing the meat quality analyses.

### 2.5. Meat Quality Analysis

The right breast meat portion from all slaughtered birds was removed from the carcass by cutting around the furcula or wishbone alongside the keel bone, and then weighed. Where applicable, sub-samples/steaks of the breast muscle were cut for various measurements.

#### 2.5.1. Breast Meat pH

The pH of the right breast meat was measured by positioning the portable meat pH meter (Hanna, HI 98163, Keysborough, VIC, Australia) 1 cm deep into the centre of the breast meat tissue and the readings recorded for all samples. The pH meter was calibrated with pH 4.01 and 7.01 standards according to the manufacturer’s instructions and cleaned using distilled water between measurements.

#### 2.5.2. Colorimetry

The calorimetric characteristics, L* (lightness), a* (red index) and b* (yellow index) of the right breast meat were measured [19] using a Konica Minolta Chromameter CR-400/410 (Thermo Fisher Scientific Pty Ltd., Waltham, MA, USA) set at d:0° (diffuse illumination/0° viewing angle; specular component included), with a standard observer angle of CIE: 2°. The colorimeter light source was a pulsed xenon lamp. Three readings, each at a different position, were taken, and the mean of the lab ordinates was automatically calculated and used in further statistical analyses. To calculate the hue and chroma to determine the precise meat colour, the following equations were used:

(1) $$\mathrm{Chroma}\;\left(C*\right)=\sqrt{\;{\left(a*\right)}^{2}+{\left(b*\right)}^{2}}$$

(2) $$\mathrm{Hue}\;\mathrm{angle}\;\left(h^{\mathrm{ab}}\right)=\tan-1\;\left(\;\frac{b*}{a*}\;\right)$$

#### 2.5.3. Water Holding Capacity

The water holding capacity (WHC) of the breast meat samples was measured using the filter paper press method (FPPM) [20]. A small sample of 1 g was cut from each of the breast meat samples and placed between two filter papers. The sample and filter paper altogether were placed between two Perspex plates and then pressed using a high-pressure (588 Newton (N) for 1 min. A digital photograph was taken of the filter paper to show the amount of water expelled from the sample. Finally, ImageJ for Mac OS X, a java-based image processing program, was used to determine the ratio of water (outer area) and meat (inner area) of the sample to calculate the WHC. In addition, the difference between the outer and inner areas was used to predict the drip loss of the samples following the equation below [20]:

(3) $$\mathrm{Water}\;\mathrm{Holding}\;\mathrm{Capacity}\;=\;\mathrm{total}\;\mathrm{water}-\mathrm{loose}\;\mathrm{water}\;\%\;\mathrm{Loose}\;\mathrm{Water}=\left(b-a\right)\times \frac{0.0084}{1}\times 100\%\;b:\;\mathrm{area}\;\mathrm{enclosed}\;\mathrm{by}\;\mathrm{the}\;\mathrm{outer}\;\mathrm{front}\;\left({\mathrm{cm}}^{2}\right)\;a:\;\mathrm{area}\;\mathrm{enclosed}\;\mathrm{by}\;\mathrm{the}\;\mathrm{inner}\;\mathrm{front}\;\left({\mathrm{cm}}^{2}\right)\;$$

#### 2.5.4. Cooking Loss

Cooking loss was calculated by subtracting the individual breast meat sample post-cooking weight from the pre-cooking weight of the breast meat sample after cooking it in a zip-lock bag in an 80 °C water-bath for 45 min. The cooked breast meat sub-samples were removed and allowed to cool down to room temperature for 10 to 15 min in a cold-water bath. The cooled cooked sub-samples were blotted dry with absorbent paper towels and weighed.

% Cooking Loss = ((weight before − weight after)/(weight before)) × 100(4)

#### 2.5.5. Warner–Bratzler Shear Force

The tenderness of the cooked breast meat samples was analysed using the Warner–Bratzler Shear Force (WBSF) test [21]. From the centre of the cooked breast sub-sample, two adjacent 1 × 1 cm, minimum of 2 cm long, meat strips were cut parallel to the muscle fibre’s orientation using pre-set scalpel blades (1 cm apart). A universal texture analyser (Instron 5543 model, 15 Stud Road Baywater, Melbourne, Victoria, Australia) equipped with a Warner–Bratzler (WB) blade was used to measure the force (N) needed to shear a strip of cooked breast meat perpendicular to the muscle fibre’s orientation. The WB blade was a 1 mm thick isosceles triangle with sides 45 mm long and a 60° cutting angle. A 2 kN load cell was used, and the crosshead speed was set at 200 mm/minute. The maximum shear force value of all cooked breast meat samples was measured in N, where a higher value indicated a tougher breast meat. A minimum of three measurements per sample was recorded, and the average was used as the WBSF reading.

#### 2.5.6. Meat Chemical Composition

The breast meat sub-samples were stored at −20 °C. Breast sub-samples were later defrosted at 4 °C for 6 h, the meat samples ground and a subsample taken for moisture determination in an oven at 105 °C for 48 h. The remaining meat samples were freeze-dried for 3 days in a freeze dryer. The dry meat sub-samples were ground again in a blender. The fat content of the meat was determined in a Soxhlet apparatus using ether as solvent [22]. The ash content of the sub-samples was determined after combustion at 550 °C for eight hours. The N content of the sub-samples was determined using a LECO CN928 Carbon/Nitrogen combustion analyser and multiplied by 6.25 to calculate crude protein content. The combustion temperature was 1100 °C, and about 0.3 g of the sub-sample was used for the analysis.

### 2.6. Gut Morphology

During the dissection, the small intestine samples were divided into the following segments: duodenum, jejunum, and ileum. Each segment was flushed with distilled water, and a small section (~1 cm length) was cut from the mid-region of each segment. Samples were immersed in 10% neutral buffered formalin solution to preserve the tissue samples for morphology analysis. The pH values from the contents of the duodenum, jejunum, ileum, and caecum segments were recorded using the same procedure described and equipment used for the meat pH readings. Fixed tissues were loaded into appropriate size cassettes for further gut histo-morphological analysis. Each fixed intestinal tissue sample was dipped in wax and a 5 mm section was cut and embedded in paraffin. Embedded intestinal segments were cut at a thickness of 6μm (Leica semi-automated RM2245 rotary microtome, Leica Microsystems, VIC, Australia) and mounted onto slides. The slides were stained by Hematoxylin and Eosin (HE), dried in an oven overnight at 37 °C, and cleared by xylene for 2 min to be scanned by light microscopy. The slides were scanned by an Aperio ScanScope XT (Leica Microsystems, VIC, Melbourne, Australia) and the villus height, crypt depth, villus width, and the number of goblet cells determined. The villus surface area and the villus height to crypt depth ratio were calculated. Villus height was measured from the tip of the villus to the crypt between two individual villi. Crypt depth was measured from the valley between the bases of the villi to the submucosa. Villus width was calculated from the mean value of the villus’ width at one-third and villus’ width at two thirds of the height of the villus. The area between the four villi was used from three cuts per sample to count the number of goblet cells. The average of the three measurements was then reported as the number of goblet cells per surface area.

### 2.7. Microbial Profile

#### 2.7.1. DNA Extraction and 16S rRNA Gene Amplicon Sequencing

Caecal content samples were collected from the sacrificed birds at 42 days using sterile scissors. Samples were immediately flash frozen in liquid nitrogen and stored in −20 freezer until DNA extraction. Caecal samples were thawed on ice, and 50 mg of each sample was transferred into sterile screw-cap tube containing sterile 0.1 and 1.0 mm zirconia beads (total weight 0.4 g; ratio 1:1). For complete genomic DNA (gDNA) extraction, microbial cells were lysed using Lysate buffer (Promega, AS1010, Madison, WI, USA) with a Qiagen TissueLyser II (Qiagen, Hilden, Germany; 30 Hz, 60 sec, 1 repetition settings). Following lysis, tubes were left for 2 min to allow the lysate to phase separate, and each sample supernatant (600 µL) was processed for gDNA extraction and purification using the Maxwell 16 blood DNA purification kit (Promega, AS1010, Madison, WI, USA) and the automated Maxwell 16 MDx instrument (Promega, Alexandria, NSW, Australia), according to the manufacturer’s instructions. Quality and quantity of extracted gDNA were assessed using a NanoDrop 1000 spectrophotometer (Thermo Scientific, Brisbane, Australia). DNA samples were sent to the Australian Genome Research Facility (AGRF Ltd., Melbourne, VIC, Australia) for amplicon sequencing.

The V3-V4 region of the 16SrRNA gene was amplified using specific primers (F: 5′-CCTAYGGGRBGCASCAG-3′, R: 5′-GGACTACNNGGGTATCTAAT-3′). The PCR condition were one cycle of 2 min at 95 °C, 30 cycles of 20 s each at 95 °C, 55 °C for 15 s, and 72 °C for 5 min, followed by one cycle of 10 min at 72 °C. PCR products (300 bp) were sequenced on Illumina MiSeq platform (AGRF, Melbourne, VIC, Melbourne, Australia).

#### 2.7.2. Bioinformatics Analysis

Raw data files were provided by AGRF as fasta files. Pair-end sequence data underwent standard demultiplexing, which reads/determines quality control, operational taxonomic units (OTU) clustering (≥97% similarity), and taxonomic classification. Microbial profiling was performed with QIIME 2 2019.7 [23]. The demultiplexed raw reads were primer trimmed and quality filtered using the CUTADAPT plugin followed by denoising with DADA2 [24]. Taxonomy was assigned to amplicon sequences variant (ASV) using the q2-featureclassifier [25] and classify-sklearn naive Bayes taxonomy classifier against SILVA v128 database [26].

Alpha-diversity analysis was performed using EstimateS v9.1.0 [27]. The OTUs with average relative abundance less than 0.005% were discarded for alpha-diversity analysis. Diversity indices of Chao1, Shannon, and Simpson were calculated, and the averages were compared between treatments. Beta-diversity analysis was performed in PAST v4.03 [28]. Bray–Curtis metric was calculated and PERMANOVA with 1000 permutations were run to compare the beta-diversity between samples and treatment groups. Principle coordinate analysis (PCoA) was performed to visualize the between group diversity. Relative abundance of microorganisms at different taxonomical levels (reported as percentage) was statistically compared between treatments using ANOVA via General Linear Model (GLM) procedure of SAS 9.4. *p* < 0.05 was used as significant threshold in Tukey’s test.

### 2.8. Statistical Analyses

The growth performance parameters, organs’ development, meat quality, and gut morphology data were analysed using Mixed Models of SAS [29], in a randomized block design with six experimental diets as class effects and blocks as random effects. Each pen was used as the experimental unit for the analysis of performance data, while the individual bird was used as the experimental unit for the analysis of other parameters. All *p* ≤ 0.05 values were deemed statistically significant. The reported least square means were separated using Fisher’s least significant differences as the post hoc test.

## 3. Results

The basal feed was mixed and supplied externally by a local feed producer based on the provided formulation. However, the feed chemical composition analyses showed some discrepancies between the calculated and analysed values for the crude protein content (Table 3), which may have affected the outcomes of the study. In addition, in the second week of the trial, there was an *Escherichia coli* outbreak in the shed which caused a higher mortality rate than expected, especially for treatments PRO-WS (11.90%) and SYN (11.90%) groups compared to NC (8.33%), PRO250 (7.14%), PC (5.95%), and PRO500 (5.95%). During the challenge, the broiler chickens were regularly checked by the veterinary services of the University of Queensland and as the infection was not severe, no medication was prescribed. Birds recovered within the next week (week 3) and the original experiment followed the original design; however, this incident has introduced a challenge to the trial which may also have affected the outcomes of the trial; this has been discussed in more detail where relevant.

### 3.1. Growth Performance, Carcass Composition, and Organ Weights

The growth performance traits (Table 4) indicated that the PRO-WS improved ADFI (*p* = 0.02) compared to PC and PRO500, but was not different when compared to NC, PRO250, and SYN. Final body weight (*p* = 0.14), and ADG (*p* = 0.14) tended to be improved by PRO-WS followed by PRO250 and SYN. The FCR was not affected by experimental treatments.

The effects of the experimental treatments on the broiler chickens’ organs relative weight are presented in Table 5. The addition of antibiotics and or probiotics in different doses into the feed, or water, and in combination with the prebiotic, did not affect the carcass weight or composition in terms of breast and abdominal fat pad weights in ratio to final BW. The relative weight of internal organs including heart, liver, bursa of Fabricius, spleen, gizzard, pancreas, and proventriculus were not affected by the experimental treatments.

### 3.2. Meat Quality

The effects of probiotic, prebiotic and synbiotic in feed or water on meat quality parameters of broiler chickens at day 42 are reported in Table 6. All meat quality traits were similar for the birds on the different treatments except for the breast colour where the breast lightness (L*) differed (*p* = 0.02) among experimental groups; birds on PRO250 had lighter breasts than birds on NC, PRO500 or PRO-WS and SYN, but were similar to PC. As pertaining to the proximate chemical composition of the breast, the moisture content was higher (*p* = 0.03) in SYN fed chickens compared to those receiving NC, PC, and PRO-WS, but similar to PRO250 and PRO500 fed broilers. The Ash content tended to be higher (*p* = 0.09) in NC followed by PRO-WS and PRO500. The breast weight and pH, water holding capacity percentage, Shear force, cooking water loss, breast’s a* and b*, Hue, moisture percentage, crude protein, and crude fat contents did not differ among all experimental treatments.

### 3.3. Gut Morphology and Excreta pH

The effects of the different experimental treatments on gut morphology traits of broiler chickens are presented in Table 7. Villus height in the duodenum was higher in PRO500 (*p* = 0.04) compared to all the other experimental groups. Crypt of the duodenum was deeper (*p* = 0.02) in PRO500 compared to NC, PRO250, PRO-WS, and SYN, but not different to PC. Duodenal villus width was wider (*p* = 0.05) in PRO500 compared to PRO250 and PRO-WS, but did no differ from NC, PC, and SYN. Villus length, VH:CD ratio and the number of goblet cells in the duodenum of the chickens were not affected by the experimental treatments. In the jejunum segment, the villus width of the chickens in PRO-WS group showed a higher (*p* = 0.03) surface area, when compared with NC and PC treatments, but was similar to PRO250, PRO500, and SYN. However, all the other studied parameters in the jejunum, including villus height, crypt depth, villus length, VH:CD ratio and number of goblet cells were similar between the different treatments. In the ileum segment of the small intestine, villus height, crypt depth, villus width, villus length and number of goblet cells were not affected by the dietary treatments, however, VH:CD ratio was significantly greater in the PRO-WS group (*p* = 0.02), compared to all the other experimental treatments.

The pH of excreta at different gut segments, including the duodenum, jejunum, ileum, and caecum of the broiler chickens at day 42, was not affected by probiotic inclusion into the feed at different doses, nor in the water, or in combination with prebiotics.

### 3.4. Microbial Profile

On average, a total of 243,294 read sequences (0.146 GB) were yielded from each caecal sample. Following sequencing quality control, a total of 203,946 sequence reads remained, of those, 199,375 and 139,650 reads were denoised and non-chimeric, respectively.

**Figure 1:**
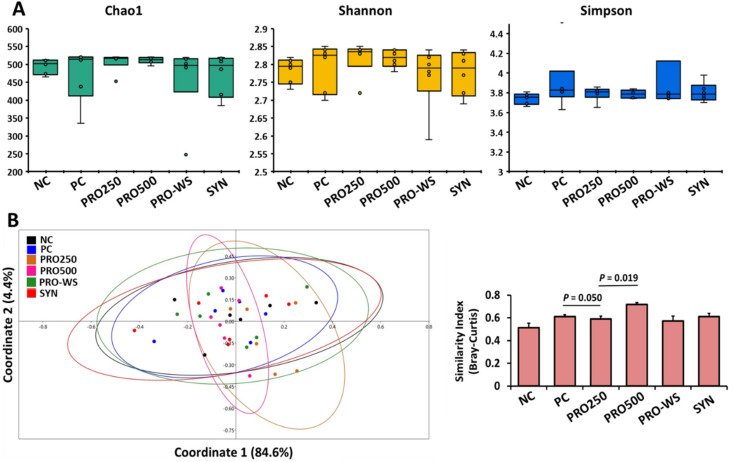
Alpha-diversity (**A**) and Beta-diversity (**B**) and comparison between different treatments fed to broiler chickens. NC: Negative control; PC: Positive control (NC plus antibiotics 200 g/t); PRO250: NC plus probiotics 250 g/t; PRO500: NC plus probiotics 500 g/t; PRO-WS: NC plus probiotics in water 0.25 g/L; SYN: NC plus probiotics 250 g/t plus prebiotics 250 g/t.

Alpha diversity metrics (Chao1, Shannon, and Simpson), which are indications of richness and evenness of the samples, are shown in Figure 1A. There were no differences (*p* > 0.05) in Chao1, Shannon, and Simpson indices between treatment groups. Beta-diversity results showed that PC (positive control with antibiotics) had less diversity (higher similarity index) compared to PRO250 (*p* = 0.050). In addition, PRO500 compared to PRO250 showed a significantly higher similarity index (*p* < 0.05), but PC and NC groups did not differ (*p* > 0.05).

**Figure 2:**
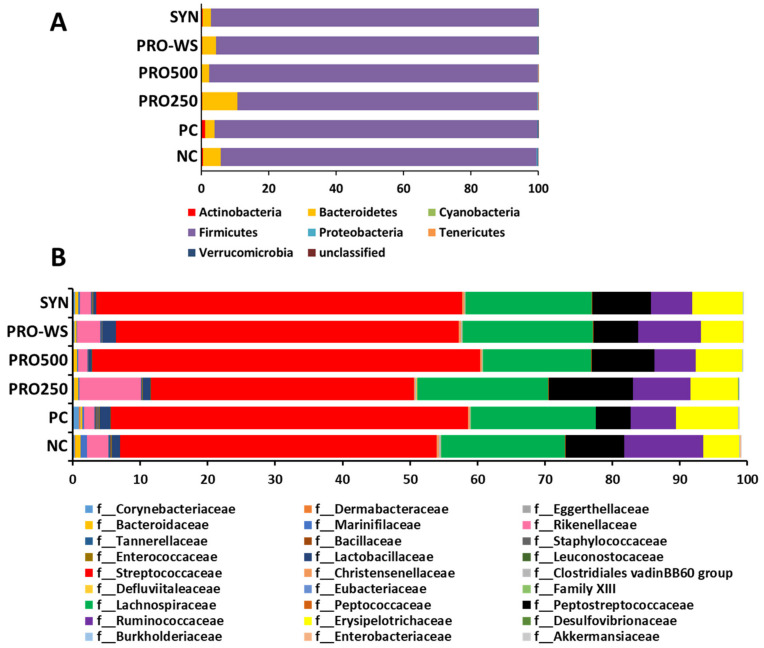
Relative abundance of OTUs at phylum (**A**) and family (**B**) levels between experimental treatments. NC: Negative control; PC: Positive control (NC plus antibiotics 200 g/t); PRO250: NC plus probiotics 250 g/t; PRO500: NC plus probiotics 500 g/t; PRO-WS: NC plus probiotics in water 0.25 g/L; SYN: NC plus probiotics 250 g/t plus prebiotics 250 g/t.

The relative abundance of different bacteria at both phylum and family levels is presented in Figure 2. Firmicutes was the most abundant phylum, followed by Bacteroidetes (Figure 2A). *Streptococcaceae, Lachnoospiraceae, Peptostreptococcaceae, Ruminococcaceae*, and *Erysipe-lotrichaceae* were the top five most abundant families (Figure 2B).

Table 8 shows the relative abundance of bacteria at both family and genus level. A Total of 88 families were identified, out of which one family differed (*p* < 0.05) and one showed a tendency (*p* = 0.565) to differ in the relative abundance between treatment groups. These two families were *Peptococcaceae* and *Tannerellaceae*, respectively. Relative abundance of *Peptococcaceae* in NC and PRO-WS groups compared to PC was higher (*p* < 0.05). There was no difference in the abundance of *Peptococcaceae* between PC and other groups (PRO250, PRO500, SYN; *p* > 0.05). As for *Tannerellaceae*, the NC group tended to have higher relative abundance compared to PRO500 (*p* = 0.057). There was no difference between the negative and positive controls.

At genus level, relative abundance of three genera was significantly affected by the treatment groups (Table 8). The first genus belongs to the family of *Peptococcaceae* and was not identified and is thus classified as “uncultured”. The abundance of *Lachnoclostridium* in the PRO-WS was higher than the PC (*p* < 0.05) treatment. As for the *Clostridiales* vadinBB60 group, the relative abundance in PRO500 was lower than the NC (*p* < 0.05).

Table 9 shows the comparison between treatments at species level. More than 400 species were identified, among which seven were affected by the treatment groups (*p* < 0.05; Table 9). Species of *Erysipelatoclostridium* were more abundant in the PC compared to the PRO500 group (*p* < 0.05). *Rusminococcaceae* species, *GCA-900066225_uncultured bacterium* (*p* < 0.05) and *GCA-900066225* (*p* < 0.03)*,* were higher in the NC group compared to SYN and PRO-WS, respectively. *Bacteroides fragilis* was more abundant (*p* < 0.05) in NC compared to the probiotic treatments (PRO250, PRO500, PRO-WS, and SYN). As for *Eubacterium* sp. Marseille-P3202, the relative abundance was higher (*p* < 0.05) in PRO-WS compared to PC.

## 4. Discussion

Probiotics are commonly added to poultry feed as a natural and green feed additive, with reported beneficial effects on growth performance, nutrient digestion, immune response, and gut morphology and microbiota [5,6,30,31]. The aim of the current study was to compare the effectiveness of probiotics added into the feed in two different doses (PRO250 & PRO500) or in water (PRO-WS), or as a combination with prebiotics (SYN) on the production efficiency and gut morphology of commercially reared chicken broilers, whilst a diet with no addition (NC) and a second diet with antibiotics (PC) would serve as control diets. The results of the current study revealed no differences between NC and PC in growth performance traits indicating the birds performed as efficient without needing antibiotics as growth promoters. It is argued that the high hygienic standards of the experimental facility and the low stocking density of the birds in the research layout do not introduce a health challenge that requires antibiotics. This argument is borne out further in that neither dose of probiotics in the feed, nor water soluble probiotics or synbiotics had any effects on the feed efficiency of the broiler chickens in the current study. However, the unexpected *E. Coli* outbreak created a challenge across the shed for all treatments and caused higher mortality rates than normal. Although the mortality rate was not statistically different among the experimental groups, birds on PC and PRO500 treatments had the lowest mortality rate (5.95%) compared to the other groups (NC: 8.33%, PRO250: 7.14%, PRO-WS: 11.90%, and SYN: 11.90%), indicating a potentially better immune response. Birds recovered after a week without any medication, and the trial followed its original plan; however, this challenge may have impacted the growth performance of birds.

Considering the overall 42-day growth performance, the PRO-WS significantly improved ADFI (*p* = 0.02) when compared to PRO500 and PC; however, PRO-WS did not differ from the NC, PRO250, and SYN treatments for these performance traits. The FCR in the current study are similar to the outcomes of a recent study [30] where no differences in production performance of birds that received probiotics in the feed or water compared to the negative and positive control groups were noted. Similarly, the addition of two different probiotic preparations, including *Bacillus coagulans* (1 g Bacillus/kg dried culture) and Lactobacillus (1 g/kg dried culture of 12 commercial Lactobacillus strains) and fructo-oligosaccharides or mannan-oligosaccharide prebiotics (each added at 5 g/kg), had no effects on the growth performance of broiler chickens [32]. In line with our findings, no significant improvement in body weight, feed intake, and FCR of broiler chickens was reported for a 5-week experimental period regardless of the route of probiotics administration [11]. However, several studies have reported improved growth performance and reduced mortality rates of broiler chickens when probiotics were supplemented into the feed [14,33]. Another study [29] reported an enhanced performance of broilers fed probiotic containing three *Bacillus* spp. and noted that among the three *Bacillus* spp., *B. coagulans* remarkably improved the growth performance. Some reports indicate that the addition of different probiotics in water improves feed intake in broilers [3,34], which is in agreement with ADFI data for PRO-WS group in the current trial. In another study, ADG and feed efficiency were enhanced for the first 3 weeks, but not for the second 3 weeks, when broilers were fed with *L. fermentum* and *S. cerevisiae* probiotics [35]. Few reports stated either no impact or a smaller benefit on the growth performance of broilers by water-based probiotics compared to those added into the feed, leaving the research question with no conclusive answer [11,33,36]. There are numerous factors contributing to the effectiveness of probiotics’ administration in broiler chickens’ diet which makes it challenging to compare different studies and assess overall outcomes. These factors include bacterial strains, stability, and viability of the species in the feed or water, administration route, basal diet ingredient composition, bird flock specification such as age, breed, and overall health status, farm or facility hygiene standards, and environmental stressors [14,30,37].

It was hypothesized that water-soluble probiotics might be more effective, as probiotic spores may remain unchanged while passing the bird’s upper GIT mainly due to the quicker transit time. In addition, the water may restrict the destruction impacts of gastric acid secretion on the microorganisms [38].

In modern intensive poultry farming systems, eggs are hatched in the hatchery facility and transferred to the grow-out sheds, in a way that the newly hatched chicken has no contact with its mother hen. Therefore, it is believed that the intestine of a newly hatched chicken is nearly sterile and gut microbiota originates from the faecal and/or environmental contaminants around the egg [39]. Recent studies, however, have reported that the egg white and embryo show a similar microbial profile and that the egg white is the source of the intestinal microbiota of the chicken embryo prior to hatching [40]. Some researchers have suggested that the colonization of the reproductive tract of mother hens by pathogenic bacteria such as *Salmonella* species is vertically transmitted to their chickens [41,42]. As a result, using probiotics in broiler breeder hen nutrition, hatchery facilities, transportation vehicles, and broiler chicken diets may be more successful in establishing the diverse and healthy gut ecosystem and improving broiler production efficiency.

Our findings showed that the probiotics in feed or water and synbiotics in feed did not affect the relative organ weights of the broiler chickens after feeding the birds for 42 days in a typical production cycle. These findings are in line with multiple studies that have used different strains of probiotics for varying durations via different routes, with no effects on relative organ weights [11,14,33,34,40,43]. Although an increase in pancreas weight after 21 days of probiotics administration has been reported [11], the reason for this increase was unknown. Other studies [44,45] reported a greater carcass and breast muscle yield when probiotics were added to the feed, which is not supported by the current study. These inconsistent results may also depend on administration level or route, basal diet composition, strains and probiotic concentrations as discussed previously.

Meat quality traits of broiler chickens were not affected by probiotics administration in feed or water for 42 days. Similar to the current study, no effects of probiotics addition to the feed or water on meat tenderness traits including water holding capacity, cooking loss, and shear force were reported [46]. It has been reported that probiotics enhance meat quality traits, including colour, oxidation stability, water holding capacity, flavour and juiciness [47,48,49,50]; however, *Bacillus subtillis*-based probiotics had negligible effects on the texture of the cooked meat. Meat colour, tenderness, and water holding capacity are important quality traits. The change in pH is one of the most significant changes that can affect meat quality characteristics which attribute to consumer acceptance [51].

The chicken breast meat lightness (L*) values can be classified as follows: lighter than normal (light, L* > 53), normal (48 < L* < 53), and darker than normal (dark, L* < 46) [51]. In the current study, the breast meat lightness was classified as lighter than normal for all experimental groups except for PRO250 and antibiotic groups. Consumers may reject chicken meat in which the quality varies from the expected normal appearance. In agreement with our results a lighter breast colour in broiler chicken fed diets supplemented with probiotics, AGPs, or the combination of probiotics and AGPs compared to the control diet has also been reported previously [51]. However, another study [46] reported a decrease in breast meat lightness of broiler chickens when fed with probiotics. There were minimal differences in the proximate chemical composition of the breast meat of the chickens fed the different dietary treatments. In this experiment, only the moisture content of the breast meat of the chickens fed PRO250, PRO500, SYN was higher than that of the NC, PC, and PRO-WS treatments. No differences in moisture, crude protein, and ash content were observed; however, a reduced fat content of the breast meat of birds fed with probiotics, AGPs, and the combination compared to a control diet have been observed [51].

In the current study, the breast pH was not affected by the dietary treatments. Overall, these results suggest that probiotic supplementation had no negative effects on meat quality traits.

The addition of probiotics has been reported to alter gut histomorphology; however, the degree of change varies depending on the strain. In the present study, intestinal morphology was improved when probiotics were added into the feed or water. Previous studies on dietary supplementation of *Bacillus* spp. probiotics including *B. subtilis* and *B. licheniformis* have reported increased VH:CD and improved function of intestinal barrier leading to a greater nutrient absorption [31,52]. Different strains of probiotics have been studied for their influence on gut histomorphology and have been found to affect the gut morphological measurements differently. A previous study on the effects of a probiotic composed of different *Bacillus* species on intestinal morphology of broiler chickens found that *B. coagulans* improved the intestinal morphometric parameters the most [31]. It has been reported that probiotics containing *Lactobacillus* spp. including *L. casei* and *L. acidophilus*, *Bifidobacterium thermophilum*, and *Enterococcus faecium* improved the villus height and decreased the crypt depth in the jejunum [53]. However, the administration of *L. johnsonii* through feed, water or litter did not change the ileal morphology of birds on day 7 and 21 [11]. Our results showed a positive influence on the morphological measurements of the small intestinal mucosa such as increased villus height, villus width, and VH:CD, suggesting that the addition of probiotics can enhance the intestinal mucosal architecture.

The data from the current study indicate no changes in the pH of the excreta in the different intestine segments of the duodenum, jejunum, ileum, and caecum of broilers when receiving probiotics with or without prebiotics in feed or water. These findings are in accordance with a previous study [11], which reported an unchanged pH load in chickens fed different probiotics and/or prebiotics. It is believed that probiotics alter the gastrointestinal pH and have a great impact on composition and function of the gut microbiome to favour an increased activity of intestinal enzymes leading to increased digestibility of nutrients. However, the effectiveness of the probiotics to reduce gut pH is variable and depends on the bacterial strain, inclusion dose, and the survival rate of the beneficial bacteria or their spore in the acidic upper GIT of the chicken. Multiple other factors can also affect gut pH such as environmental conditions of the poultry shed, feed stuff sanitation and feed processing hygiene at the feed mill [54,55].

The GIT of broilers is a favourable environment for the growth of diverse microbiota. The microbial profile of the gut is, however, dynamic and factors such as age, especially the early stages of life, genotype, farming conditions/environment, and diet/feed additives all influence the makeup of the chicken’s gut microbiota [56]. Various stressors can alter the gut microbiota composition, leading to dysbiosis, and can consequently impact the functionality of the intestine, e.g., increased permeability, increased risk of bacterial infection, sepsis, inflammation, and slower digestion [57]. A study of the diversity of gut flora in the ileum and caeca of broiler chickens when fed with a corn–soybean meal diet with no additives revealed that ileum is mostly colonized by *Lactobacillus* (70%), with the rest of the bacteria belonging to *Clostridiaceae* (11%), *Streptococcus* (6.5%), and *Enterococcus* (3.5%) families (6.5%). Conversely, in the caecum, *Clostridiaceae* (65%) were the most abundant group followed by *Fusobacterium* (14%), *Lactobacillus* (8%), and *Bacteroides* (6%) [58]. Probiotics can enhance the balance of microbiota by competitive exclusion of pathogens through occupying binding sites and receptors on the intestinal mucosa and suppression of the growth of other microbes by producing antimicrobial agents [6,59].

Although many papers have been published on probiotics in broiler chicken nutrition, literature regarding probiotics administration in feed or water and its effects on broiler intestinal microbiota has only been studied sparsely using 16S rRNA gene amplicon sequencing and molecular methods. The findings of the current study revealed no changes in microbial alpha-diversity across all treatments, with no significant prevalence of any bacterial species being linked to any experimental treatment. These findings were similar to that of Zhu and co-workers [60], who reported no significant effects of antibiotic or heat-inactivated compound probiotics treatments on alpha-diversity, including observed species, Chao 1, Shannon index, Simpson index, Goods coverage, ACE, and PD whole tree (*p* > 0.05). In the present investigation, the beta-diversity of the microbial profile of the chicken’s caecum content was slightly reduced in the PC group compared to other treatments, indicating a potentially reduced microbial diversity when birds received antibiotics in feed at subtherapeutic levels; however, this needs to be verified in a repeated experiment, possibly with a more pathogenic challenging environment. Comparable results were also reported by Zhu and co-workers [60] where the beta-diversity index of cecal microbiota was significantly higher than that of PC group at the same sampling timepoints. Accordingly, Gao et al. [61] reported opposing effects of probiotic and antibiotic administrations on the age-dependent maturation of intestinal microbiota. The probiotic treatment showed an early-maturing trend, reaching a plateau at day 15 and indicating an accelerated maturation of the gut microbiota, whereas antibiotics showed a delayed microbiota development, and the beta-diversity of intestinal microbiota changed more heavily in the antibiotic group from day 1 to 42. This indicates that antibiotics destroy a portion of the gut microflora, causing diminished levels of microbial diversity.

The most abundant phylum was *Firmicutes*, followed by *Bacteroidetes*. There was no significant difference between the treatments regarding relative abundance of the phyla. At the family level, *Peptococcaceae* were significantly reduced by the antibiotic and not by the probiotic treatments when administered via water and not through feed. This could indicate an interaction between the microbial community and the administration method. At the genus level, three genera were identified that were significantly affected by the treatment groups. Genus *Lachnoclostridium* was significantly increased by PRO-WS compared to the antibiotic positive control. This genus includes bacteria from several clostridial clusters which are known to have anti-inflammatory effects and play important role in homeostasis [61]. Gao et al. [62] found eight genera that were significantly changed on day 28; however, no genera were found significantly changed on day 42. In the present study, the relative abundance at species level showed seven species that were significantly affected by the treatment groups. However, only *Bacteroides fragilis* could be detected to the species level. The remaining were unknown. *Bacteroides fragilis* was significantly higher in NC compared to PRO250, PRO500, PRO-WS, and SYN, but not different from the PC. This indicates that the tested probiotic product, reduced the abundance of this organism in the chicken’s cecum. *Bacteroides fragilis* which accounts for 0.5% of the colonic flora in the human, is the most isolated pathogen [63]. In chickens, *B. fragilis* species has a different pattern than in the human and showed high antimicrobial resistance [64]. This might be the reason why the antibiotic group did not reduce this species compared to the negative control. However, these results indicate that the probiotic used in this study has the potential to reduce the risk of *Bacteroides fragilis*. This could also have health implications for humans since *Bacteroides fragilis* operate as an amphibiotic organism in the colon, and as a result, they are key markers and sources of antimicrobial resistance genes during endogen infections [64]. Meat chickens are an important source of protein for humans, but they are also a source of antimicrobial resistance genetic determinants that can be passed to other bacterial species in the human digestive tract following ingestion [65].

## 5. Conclusions

In the present study, the positive and negative control groups performed similarly, which indicates an ideal environment with insufficient challenges where birds did not need any antibiotics. It is recommended to test the probiotics in a more challenging environment to evaluate the efficacy of the positive control and other treatments. Although an unexpected *E. coli* outbreak introduced a mild health challenge to the study on the second week, however, birds recovered without any medication; it is assumed that this environmental challenge was not severe enough to test the efficacy of the treatments. Nonetheless, based on the outcomes of this study, probiotic supplementation via feed or water could partially enhance the production performance and gut morphometry parameters in broiler chickens; however, the lack of consistent improvement in the different studied parameters from a specific treatment makes it difficult to conclude on a dose or administration route. The results obtained from the current experiment are evidence that supplementation of specific probiotics mixtures can modulate the microbiota that colonizes the gut and increase the diversity of the bacterial community where antibiotics reduced it. In addition, the relative abundance of *Bacteroides fragilis*, which is a marker of bacterial resistance, was reduced in probiotic-containing treatments. Further research is required to study probiotics supplementation and its effects on the bird’s immune response in challenge with non-resistant pathogens.

## Figures and Tables

**Table 1:** Experimental treatments’ specification fed to broiler chickens for 42 days.

Diet Code	Diet Short Description	Diet Specification
NC	NC	Negative Control: no Probiotic, no Antibiotic
PC	PC (NC + 200 g/t Virginiamycin)	Negative Control + Antibiotic as Growth Promoter
PRO250	NC + 250 g/t Probiotic	Negative Control + Probiotics 250 g/t
PRO500	NC + 500 g/t Probiotic	Negative Control + Probiotics 500 g/t
PRO-WS	NC + 0.25 g/L Probiotic in Water Supplied (WS)	Negative Control + Probiotics in water 0.25 g/L
SYN	NC + 250 g pre + 250 g pro	Negative Control + Probiotics 250 g/t + Prebiotics 250 g/t

**Table 2:** Different Bacillus strains and specifications in Natupro^®^ probiotic.

Probiotic Strains	Specification
*Bacillus subtilis*	1.5 × 10^8^ CFU/g
*Bacillus licheniformis*	1.5 × 10^8^ CFU/g
*Bacillus amyloliquefaciens*	3 × 10^8^ CFU/g

**Table 3:** Ingredient composition, calculated and analysed nutrients composition of the basal diet ^1^.

Diet Composition	Grow-Out Phases		
	Starter (1–14 Days)	Grower (15–28 Days)	Finisher (29–42 Days)
Ingredient Composition (%)			
Wheat	40.00	40.00	45.00
Corn	20.46	20.00	21.02
Soybean meal	29.82	29.37	23.80
Soybean oil	4.27	5.89	5.84
L-Lysine HCL	0.50	0.35	0.34
DL-Methionine	0.41	0.34	0.31
L-Threonine	0.26	0.17	0.16
Limestone	1.52	1.38	1.26
Mono-Calcium Phosphate	1.74	1.51	1.32
Sodium bicarbonate	0.32	0.24	0.25
Salt	0.15	0.20	0.20
Vitamin—trace Mineral Premix	0.50	0.50	0.50
Coccidiostat	0.05	0.05	0.00
Calculated Nutrients Composition			
ME ^2^ (MJ/kg)	11.92	12.34	12.55
Crude protein (%)	21.00	20.50	18.50
SID ^3^ Lysine (%)	1.28	1.15	1.02
SID Met + Cys (%)	0.95	0.87	0.80
SID Threonine (%)	0.86	0.77	0.68
Calcium (%)	0.96	0.87	0.78
Avail. Phosphorous (%)	0.48	0.44	0.39
Sodium (%)	0.16	0.16	0.16
Potassium (%)	0.90	0.89	0.79
Chloride (%)	0.23	0.23	0.23
Analysed Nutrients Composition			
Dry matter (%)	89.66	90.80	90.93
Crude protein (%)	20.52	21.84	19.30
Ash (%)	8.14	7.25	7.25

^1^ Calculated nutrients composition is on “as is” basis and analysed nutrient composition is on “DM” basis. ^2^ ME: metabolisable energy. ^3^ SID: standardised ileal digestible.

**Table 4:** Effects of probiotic, prebiotic and symbiotic in feed or water on growth performance of broiler chickens.

Parameters ^1^	Experimental Diets ^2^						SEM ^3^	*p* Value
	NC	PC	PRO250	PRO500	PRO-WS	SYN		
FBW (g)	2365	2326	2389	2265	2405	2377	39	0.14
ADG (g)	55.32	54.40	55.92	52.96	56.32	55.63	1.0	0.14
ADFI (g)	93.07 ^ab^	90.65 ^bc^	92.63 ^ab^	88.27^c^	96.0 ^a^	92.44 ^ab^	1.54	0.02
FCR-c (g)	1.57	1.56	1.55	1.57	1.59	1.56	0.02	0.85
Mortality (%)	8.33	5.95	7.14	5.95	11.90	11.90	3.54	0.65

^1^ FBW: body weight; ADG: average daily gain; ADFI: average daily feed intake; FCR-c: mortality-corrected feed conversion ratio; ^2^ NC: Negative Control; PC: Positive Control: NC + 200 g/t Virginiamycin as Antibiotic Growth Promoter (AGP); PRO250: NC + 250 g/t Probiotic; PRO500: NC + 500 g/t Probiotic; WS: NC + 0.25g /L Probiotic in Water Supplied; and SYN: NC + 500 g/t Synbiotic (composed of a mix e of 250 g Probiotic + 250 g Prebiotic/t of feed). ^3^ SEM: standard error of means. ^a–c^ Means with different superscripts differ (*p* ≤ 0.05).

**Table 5:** Effects of probiotic, prebiotic and symbiotic in feed or water on broiler chickens’ organs relative weight to final body weight at day 42.

Parameters	Experimental Diets ^1^						SEM	*p* Value
	NC	PC	PRO250	PRO500	PRO-WS	SYN		
Relative Organ weight ^2^								
Carcass Weight	724.7	728.5	726.6	713.7	730.1	718.4	9.92	0.84
Breast Muscle Weight, (g)	245.0	229.4	234.9	233.6	242.0	236.0	5.57	0.35
Heart, (g)	3.86	4.09	4.18	3.82	4.33	4.02	0.16	0.19
Liver, (g)	16.69	16.39	17.93	16.38	16.27	16.0	0.73	0.47
Bursa of Fabricius, (g)	1.73	1.88	1.73	1.89	1.92	1.64	0.17	0.81
Spleen, (g)	0.85	1.03	0.94	0.95	1.09	0.97	0.06	0.09
Gizzard, (g)	21.25	24.22	22.14	22.22	22.27	22.41	1.12	0.58
Pancreas, (g)	1.79	2.06	2.01	1.83	1.88	1.85	0.09	0.24
Abdominal Fat, (g)	4.85	6.12	4.69	3.88	5.50	3.95	0.77	0.19
Proventriculus, (g)	4.99	5.58	4.83	5.00	5.22	5.19	0.35	0.73

^1^ NC: Negative Control; PC: Positive Control: NC + 200 g/t Virginiamycin as Antibiotic Growth Promoter (AGP); PRO250: NC + 250 g/t Probiotic; PRO500: NC + 500 g/t Probiotic; WS: NC + 0.25 g /L Probiotic in Water Supplied; and SYN: NC + 500 g/t Synbiotic (composed of a mix e of 250 g Probiotic + 250 g Prebiotic/t of feed). SEM: Standard error of means. ^2^ Relative weight = absolute weight (g)/body weight (kg).

**Table 6:** Effects of probiotic, prebiotic and symbiotic in feed or water on meat quality parameters of broiler chickens at day 42.

Parameters	Experimental Diets ^1^						SEM ^2^	*p* Value
	NC	PC	PRO250	PRO500	PRO-WS	SYN		
Breast Meat Weight (% of BW)	25.4	22.9	23.5	23.4	24.2	23.6	0.56	0.35
Breast Meat pH	5.74	5.76	5.73	5.69	5.80	5.73	0.04	0.41
Water Holding Capacity %	68.00	68.97	69.53	69.64	68.71	68.92	0.61	0.42
Shear Force (N)	22.34	20.45	21.25	21.42	20.37	22.58	2.87	0.86
Cooking Water Loss (%)	21.93	19.73	19.83	21.08	21.35	21.33	1.98	0.51
Breast Meat L*	53.71 ^ab^	52.43 ^bc^	50.77 ^c^	54.94 ^a^	53.12 ^ab^	53.03 ^ab^	0.95	0.02
Breast Meat a*	2.51	2.64	2.56	2.49	2.73	2.38	0.35	0.98
Breast Meat b*	5.75	5.85	5.26	6.24	5.59	5.57	0.38	0.59
Hue (◦)	66.22	65.75	62.94	69.39	64.56	67.22	2.89	0.60
Moisture %	73.34 ^b^	74.35 ^b^	74.71 ^ab^	74.62 ^ab^	74.47 ^b^	75.01 ^a^	0.16	0.03
Crude Protein % (as is)	22.50	22.78	22.69	22.50	22.69	22.36	0.22	0.77
Crude Fat % (as is)	1.76	1.60	1.51	1.59	1.57	1.46	0.09	0.29
Ash % (as is)	1.59	1.54	1.53	1.55	1.56	1.48	0.04	0.09

^1^ NC: Negative Control; PC: Positive Control: NC + 200 g/t Virginiamycin as Antibiotic Growth Promoter (AGP); PRO250: NC + 250 g/t Probiotic; PRO500: NC + 500 g/t Probiotic; WS: NC + 0.25g /L Probiotic in Water Supplied; and SYN: NC + 500 g/t Synbiotic (composed of a mix e of 250 g Probiotic + 250 g Prebiotic/t of feed). ^2^ SEM: Standard error of means. ^a–c^ Means with different superscripts differ (*p* ≤ 0.05).

**Table 7:** Gut morphological parameters of broilers fed with different experimental diets at day 42.

Parameter	Experimental Diets ^1^						SEM ^2^	*p* Value
	NC	PC	PRO250	PRO500	PRO-WS	SYN		
Duodenum								
Villus height (µm)	1222 ^b^	1208 ^b^	1101 ^b^	1474 ^a^	1170 ^b^	1113 ^b^	95.18	0.04
Crypt depth (µm)	51.00 ^b^	57.71 ^ab^	48.66 ^b^	68.12 ^a^	48.58 ^b^	55.87 ^b^	5.21	0.02
Villus width (µm)	160.0 ^a^	152.7 ^ab^	149.1 ^b^	184.4 ^a^	126.8 ^c^	164.1 ^a^	13.17	0.05
Villus length (µm)	62.92	64.22	60.38	59.58	62.55	62.30	1.78	0.48
VH:CD ^3^	26.96	21.98	23.75	22.54	26.82	21.10	3.02	0.49
No. of goblet cells	29.0	23.25	24.92	26.67	24.33	23.50	2.34	0.50
Duodenum pH	6.0	6.09	6.01	6.07	6.05	6.06	0.05	0.72
Jejunum								
Villus height (µm)	598	644	642	691	630	647	47.45	0.85
Crypt depth (µm)	43.37	44.21	44.06	52.87	42.47	38.14	3.76	0.16
Villus width (µm)	99.55 ^c^	107.28 ^bc^	127.52 ^ab^	130.04 ^ab^	146.8 ^a^	132.88 ^ab^	10.73	0.03
Villus length (cm)	53.33	45.40	48.87	44.50	47.43	45.75	2.91	0.30
VH:CD ^2^	14.26	13.36	16.53	14.26	16.12	17.47	1.93	0.62
No. of goblet cells	15.33	17.05	13.67	15.92	14.50	15.75	1.31	0.43
Jejunum pH	5.83	6.03	5.95	5.92	5.91	5.94	0.07	0.43
Ileum								
Villus height (µm)	566	490	539	533	574	512	39.29	0.52
Crypt depth (µm)	44.27	44.92	46.45	51.87	38.68	45.84	3.40	0.19
Villus width (µm)	123.94	104.44	139.77	117.56	121.12	117.66	9.64	0.14
Villus length (cm)	78.75	81.17	70.75	74.50	77.54	78.92	3.01	0.15
VH:CD ^2^	13.35 ^b^	11.53 ^b^	12.24 ^b^	11.40 ^b^	16.85 ^a^	11.27 ^b^	1.47	0.04
No. of goblet cells	15.03	12.67	15.83	15.58	17.25	14.50	1.17	0.09
Ileum pH	6.23	6.09	6.09	6.30	6.30	6.43	0.17	0.41
Caecum								
Caecum pH	5.96	5.93	5.99	5.87	6.01	5.88	0.09	0.85

^1^ NC: Negative Control; PC: Positive Control: NC + 200 g/t Virginiamycin as Antibiotic Growth Promoter (AGP); PRO250: NC + 250 g/t Probiotic; PRO500: NC + 500 g/t Probiotic; WS: NC + 0.25 g /L Probiotic in Water Supplied; and SYN: NC + 500 g/t Synbiotic (composed of a mix e of 250 g Probiotic + 250 g Prebiotic/t of feed). ^2^ SEM: Standard error of means. ^3^ VH:CD: villus height to crypt depth ratio. ^a–c^ Means with different superscripts differ (*p* ≤ 0.05).

**Table 8:** Relative abundance (%) of different bacteria at Family and Genus levels in caecum content of the chickens fed experimental treatments.

Level	Name	Experimental Diets ^1^							
		NC	PC	PRO250	PRO500	PRO-WS	SYN	SEM	*p* Value
Family									
*Bacteria- Firmicutes- Clostridia-* *Clostridiales_Peptococcaceae*		0.093 ^a^	0.034 ^b^	0.074 ^ab^	0.065 ^ab^	0.102 ^a^	0.061 ^ab^	0.013	0.015
*Bacteria-Bacteroidetes-Bacteroidia-Bacteroidales-Tannerellaceae*		0.279	0.170	0.096	0.075	0.137	0.110	0.030	0.057
Genus									
*Bacteria_Firmicutes_Clostridia_Clostridiales_Peptococcaceae_* uncultured		0.091 ^a^	0.033 ^b^	0.073 ^ab^	0.065 ^ab^	0.101 ^a^	0.061 ^ab^	0.013	0.017
*Bacteria_Firmicutes_Clostridia_Clostridiales_Lachnospiraceae_Lachnoclostridium*		0.096 ^ab^	0.067 ^b^	0.106 ^ab^	0.116 ^ab^	0.162 ^a^	0.124 ^ab^	0.017	0.019
*Bacteria_Firmicutes_Clostridia_Clostridiales_Clostridiales* vadinBB60 group_gut metagenome		0.0030 ^a^	0.0007 ^ab^	0.0008 ^ab^	0.000 ^b^	0.0007 ^ab^	0.0005 ^ab^	0.0006	0.072

^1^ NC: Negative control; PC: Positive control (NC plus antibiotics 200 g/t); PRO250: NC plus probiotics 250 g/t; PRO500: NC plus probiotics 500 g/t; PRO-WS: NC plus probiotics in water 0.25 g/L; SYN: NC plus probiotics 250 g/t plus prebiotics 250 g/t. ^a,b^ Means with different superscripts differ (*p* ≤ 0.05).

**Table 9:** Relative abundance (%) of different bacteria at Species levels in caecum content of the chickens fed experimental treatments.

Name	Experimental Diets ^1^							
	NC	PC	PRO250	PRO500	PRO-WS	SYN	SEM	*p* Value
Species								
*Bacteria_Firmicutes_Erysipelotrichia_Erysipelotrichales_ Erysipelotrichaceae_Erysipelatoclostridium*	0.058 ^ab^	0.12 ^a^	0.061 ^ab^	0.046 ^b^	0.053 ^ab^	0.077 ^ab^	0.018	0.050
*Bacteria_Firmicutes_Clostridia_Clostridiales_Ruminococcaceae_GCA-900066225*	0.011 ^a^	0.001 ^ab^	0.004 ^ab^	0.002 ^ab^	0.000 ^b^	0.003 ^ab^	0.002	0.030
*Bacteria_Firmicutes_Clostridia_Clostridiales_Ruminococcaceae_GCA-900066225_uncultured bacterium*	0.032 ^a^	0.009 ^ab^	0.013 ^ab^	0.009 ^ab^	0.015 ^ab^	0.005 ^b^	0.005	0.049
*Bacteria_Firmicutes_Clostridia_Clostridiales_Peptococcaceae_uncultured_uncultured bacterium*	0.086 ^ab^	0.031 ^b^	0.071 ^ab^	0.062 ^ab^	0.098 ^a^	0.056 ^ab^	0.013	0.018
*Bacteria_Firmicutes_Clostridia_Clostridiales_Lachnospiraceae_ Lachnospiraceae NC2004 group_uncultured bacterium*	0.037 ^ab^	0.028 ^ab^	0.031 ^ab^	0.064 ^a^	0.035 ^ab^	0.006 ^b^	0.013	0.047
*Bacteria_Bacteroidetes_Bacteroidia_Bacteroidales_Bacteroidaceae_Bacteroides_Bacteroides fragilis*	0.280 ^a^	0.131 ^ab^	0.093 ^b^	0.036 ^b^	0.071 ^b^	0.088 ^b^	0.035	0.0008
*Bacteria_Firmicutes_Clostridia_Clostri iales_Lachnospiraceae_ Lachnoclostri ium_Eubacterium sp. Marseille-P3202*	0.092 ^ab^	0.067 ^b^	0.106 ^ab^	0.116 ^ab^	0.162 ^a^	0.124 ^ab^	0.017	0.018

^1^ NC: Negative control; PC: Positive control (NC plus antibiotics 200 g/t); PRO250: NC plus probiotics 250 g/t; PRO500: NC plus probiotics 500 g/t; PRO-WS: NC plus probiotics in water 0.25 g/L; SYN: NC plus probiotics 250 g/t plus prebiotics 250 g/t. ^a,b^ Means with different superscripts differ (*p* ≤ 0.05).

## Data Availability

The data presented in this study are available on request from the corresponding author.
